# Erratum to: Comorbidities of Atopic Dermatitis: Beyond Rhinitis and Asthma

**DOI:** 10.1007/s13671-017-0196-3

**Published:** 2017-08-24

**Authors:** Yuki M. F. Andersen, Alexander Egeberg, Lone Skov, Jacob P. Thyssen

**Affiliations:** Department of Dermatology and Allergy, Herlev and Gentofte Hospital, University of Copenhagen, Kildegårdsvej 28, 2900 Hellerup, Denmark

**Erratum to: Curr Derm Rep (2017) 6:35–41**

10.1007/s13671-017-0168-7

The article “Comorbidities of Atopic Dermatitis: Beyond Rhinitis and Asthma”, written by Yuki M.F. Andersen, Alexander Egeberg, Lone Skov and Jacob P. Thyssen, was originally published Online First without open access. After publication in volume 6, issue 1, page 35–41 the author decided to opt for Open Choice and to make the article an open access publication. Therefore, the copyright of the article has been changed to © The Author(s) 2017 and the article is forthwith distributed under the terms of the Creative Commons Attribution 4.0 International License (http://creativecommons.org/licenses/by/4.0/), which permits use, duplication, adaptation, distribution and reproduction in any medium or format, as long as you give appropriate credit to the original author(s) and the source, provide a link to the Creative Commons license, and indicate if changes were made.

